# The Administrative Prevalence of Multiple Sclerosis in Greece on the Basis of a Nationwide Prescription Database

**DOI:** 10.3389/fneur.2020.01012

**Published:** 2020-09-29

**Authors:** Christos Bakirtzis, Eleni Grigoriadou, Marina Kleopatra Boziki, Evangelia Kesidou, Spyridon Siafis, Theodoros Moysiadis, Dimitra Tsakona, Eleftherios Thireos, Ioannis Nikolaidis, Chrysa Pourzitaki, Dimitrios Kouvelas, Georgios Papazisis, Dimitrios Tsalikakis, Nikolaos Grigoriadis

**Affiliations:** ^1^Multiple Sclerosis Center, B' Department of Neurology, Aristotle University of Thessaloniki, Thessaloniki, Greece; ^2^Department of Clinical Pharmacology, Aristotle University of Thessaloniki, Thessaloniki, Greece; ^3^Department of Psychiatry and Psychotherapy, School of Medicine, Technical University of Munich, Munich, Germany; ^4^Institute of Applied Biosciences, Center for Research and Technology Hellas, Thessaloniki, Greece; ^5^Athens Medical Society, Athens, Greece; ^6^Department of Informatics and Telecommunications Engineering, University of Western Macedonia, Kozani, Greece

**Keywords:** prevalence, multiple sclerosis, Greece, epidemiology, prescription

## Abstract

**Objective:** To estimate current prevalence of multiple sclerosis (MS) in Greece using administrative data from the nationwide medicine prescription database.

**Methods:** Prescription records of a 24-month period (June 2017–May 2019) were analyzed in order to identify cases of MS. Sex, age, and place of residence were recorded for each identified case. Prevalence of MS was calculated based on the updated records of the Greek population according to Hellenic Statistical Authority.

**Results:** The 2-year cumulative period prevalence of MS was estimated to 197.8 per 100,000 (95% CI 197.6–198.0). In total, 21,218 patients (65.8% female) were identified. During this period, the prevalence of MS was 138.7 per 100,000 (95% CI 138.4–139.0) in men and 253.6 per 100,000 (95% CI 253.3–254.1) in women. Prevalence was higher in the 45–49 age group in both sexes. Analysis of the place of residence revealed higher prevalence in the Attica region and Western Greece while lower prevalence was observed in Northern Greece. No north–south latitude gradient was detected. Point prevalence on 1 January 2019 was calculated to 188.9 per 100,000 (95% CI 188.7–189.1). Regarding treatment, 73.1% of the identified cases received at least once a Disease Modifying Drug.

**Conclusions:** According to this national-level study conducted in Greece, estimated prevalence of MS was found to be similar to those of other European countries. Heterogeneity of MS prevalence across the country was observed and needs further investigation.

## Introduction

Multiple sclerosis (MS) is a chronic autoimmune and neurodegenerative disease that affects about 2.3 million people worldwide (1). This chronic disease is one of the most common causes of disability in young populations (2). In Europe, prevalence of MS is rising (3); however, geographical variations may be observed (4, 5). It remains unclear whether this heterogeneity can be attributed exclusively to genetic and environmental factors, since access to healthcare and insurance policies varies across countries and may influence the results of prevalence studies performed on this continent.

In Greece, epidemiological data regarding MS are scarce; however, a number of previous studies have suggested an increment of prevalence from 10.2 per 100,000 in 1984 (6), to 29.5 per 100.000 in 1990 (7), 38.9 per 100,000 in 1999 (8), and more than 100 per 100,000 according to two studies performed in 2006 and in 2008 respectively (9, 10). All relevant studies were regional and applied various diagnostic criteria (Table 1). Regarding annual incidence rate, an increase from 2.71/100,000 in 1989 to 10.73/100,000 in 2006 has been reported by one study (9), while another one demonstrated the rise of incidence rates in the urban areas of the island of Crete, located in south Greece, from 2.1 per 100,000 in 1984, to 7 per 100,000 in 2008 (10). As of 2008, no new epidemiological data for Greek people with MS exist.

**Table 1:** Previous studies regarding prevalence of multiple sclerosis in Greece.

**Study**	**Region**	**Prevalence (/100,000)**	**Source of medical records**	**Range of years**	**Methodology used**	**Diagnostic criteria**
Vasilopoulos (6)	Attica	10.2	1 clinic	1964–1983	Extrapolation according to ALS global prevalence	Not mentioned
Milonas et al. (7)	Northern Greece	29.5	4 clinics and 18 private offices	1970–1984	Point prevalence on 31 December 1984	Poser
Piperidou et al. (8)	Evros	38.9	1 clinic	1974–1999	Point prevalence on 31 December 1999	Poser
Papathanasopoulos et al. (9)	Western Greece	119.6	1 clinic	1984–2006	Point prevalence on 31 December 2006	Poser and McDonald
Kotzamani et al. (10)	Crete	108	All neurologists of Crete	1980–2008	Point prevalence on 31 December 2008	McDonald

According to the Atlas of MS (1), last updated in 2013, Greece belongs to the group of countries presenting prevalence of the disease somewhere between 60 and 100 per 100,000; this information may be outdated, though. Since the landscape of MS diagnosis and treatment is rapidly evolving, newer national-level prevalence studies are needed in order to accurately calculate prevalence of the disease (4). Patient registries and health claims data may therefore be used in order to estimate the prevalence of the disease within a country (11, 12). Although these administrative data are primarily collected for reimbursement, they may also be used for epidemiological studies, since they are uniformly collected and represent a large amount of the population studied (13).

In the absence of a nationwide registry for people with MS (PwMS), we aimed to estimate current prevalence of MS in Greece using data from the nationwide prescription database. This study was performed after permission for use of pseudoanonymized and coded prescription data by IDIKA S.A.^1^, a non-profit government organization responsible for the digital health data in Greece, in accordance with the national legislations on personal data and with the ethical standards laid down in the 1964 Declaration of Helsinki and its later amendments.

## Methods

Greece is a European country that lies in the Eastern Mediterranean Sea. The country's territory includes 132,049 Km^2^ located between 34.8^o^ and 41.7^o^ N latitudes and 19.3^0^ and 29.6^0^ W longitudes. The country's population is estimated to 10.724.599 on 1 January 2019, according to Hellenic Statistical Authority (ELSTAT), which is the national statistical service^2^.

Currently, diagnosis of MS in Greece is based on McDonald criteria and their revisions (14), according to the guidelines of Hellenic Academy of Neuroimmunology (HELANI), which have been adopted by the Greek Ministry of Health (15). Up to now, disease modifying drugs (DMDs) are fully reimbursed (100%) to all Greek people with MS (PwMS). Some agents for symptomatic treatment such as baclofen and fampridine are also fully covered (100%), whereas others are partially reimbursed (90 or 75%, depending on the substance). The National Organization for the Provision of Health Services (EOPYY) is the largest national health-care provider^3^, reimbursing health expenses for almost the entire Greek population. Medical expenses of ~200,000–300,000 citizens (mainly permanent military personnel, their family members, and people without social security number) are reimbursed by authorities other than EOPYY, thus corresponding to ~1.8–2.7% of the Greek population ^1^^,^^4^. EOPYY reimburses drug prescriptions that are performed through a nationwide digital medicine prescription database^5^, which runs under surveillance of IDIKA S.A., on behalf of the Greek Ministry of Health. All prescriptions are registered under the International Classification of Diseases v. 10 (ICD-10) coding system. The code G35 is assigned to MS. This code is used by all clinicians in order to prescribe DMDs and agents for MS symptomatic treatment in Greece, whereas an additional medical report, signed by the treating physician, is also mandatory and a prerequisite for the processing of each prescription. Information such as type of MS, disease duration, and EDSS were not included in this prescription database during the study period. Therefore, the IDIKA S.A. prescription database may currently not regarded as an MS disease registry. Such national MS disease registry is currently under construction and is expected to include thorough information regarding clinical and laboratory disease parameters.

Two independent researchers analyzed prescription data from the nationwide prescription database of IDIKA S.A., dating from June 1, 2017, to May 31, 2019, and identified MS cases using the ICD-10 code for MS (G35). Criterion for the case identification was ≥ 1 MS specific drug prescription (DMD or symptomatic) in this 24-month period and the analysis was performed on the basis of the active substances. Prescriptions not executed in pharmacies were excluded from the study analysis. We then extracted data regarding age, sex, and place of residence for each individual case. All discrepancies between the two independent analyses were reviewed on a case by case basis. Finally, a third researcher validated the results by conducting a third blinded analysis. In addition, the most recent data derived from ELSTAT, regarding Greek population, were used. In brief, current Greek population was calculated based on the population census of 2011 adjusted to January 1, 2019, according to birth and death records as well as official immigration data. Finally, age, sex, and regional prevalence of the disease were calculated.

In order to explore the potential contribution of a north–south latitude gradient, the latitudinal coordinates of each region were used. After examining for normality, prevalence rates of each region were compared with the corresponding latitudes using Pearson's correlation. Level of significance was set at *p* < 0.05. Statistical analysis was performed using IBM® SPSS software 25.0 (Armong, NY).

## Results

**Figure 1:**
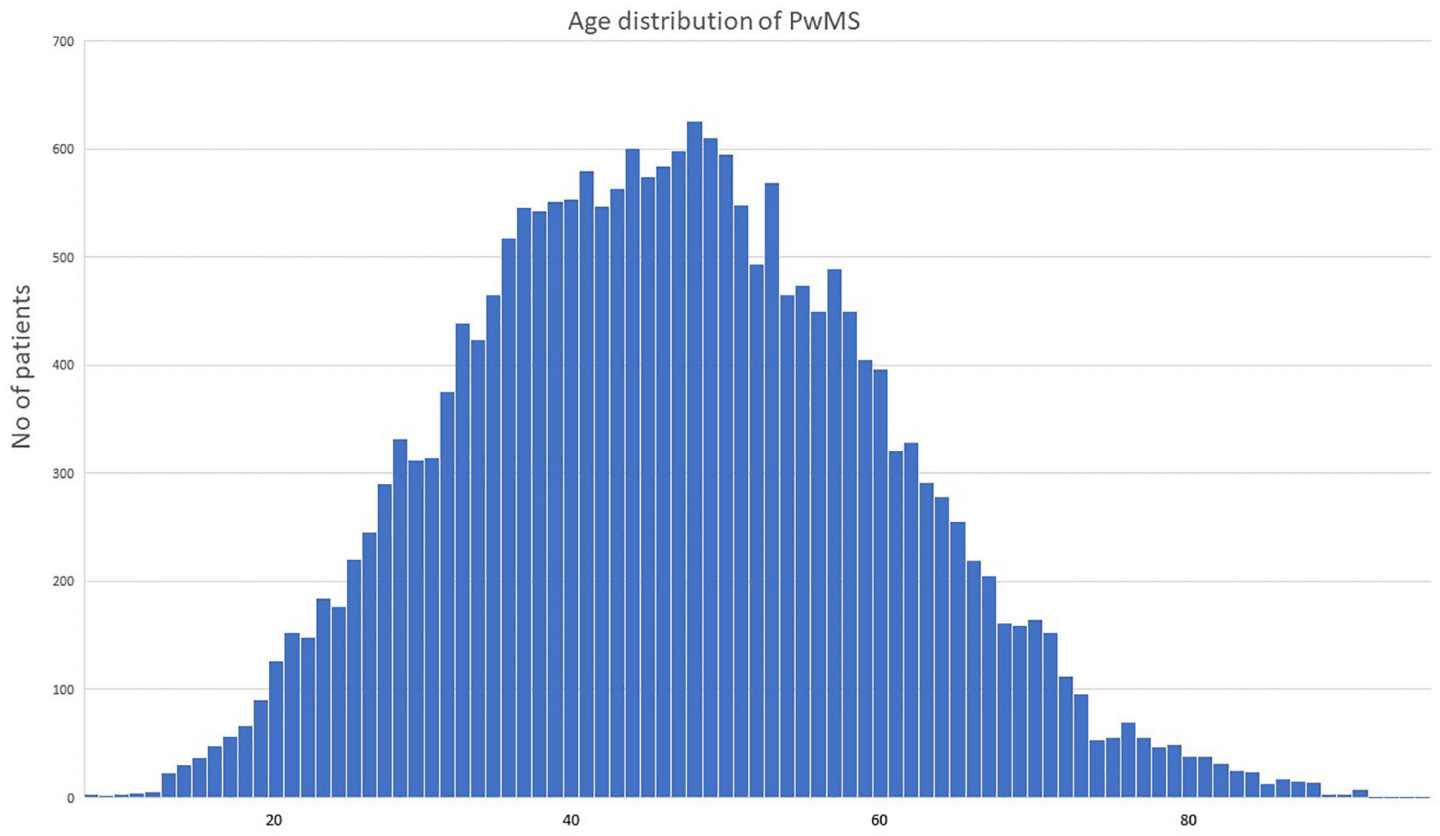
Age distribution of people with multiple sclerosis in Greece during the study period. PwMS, people with multiple sclerosis; age is indicated in years.

**Figure 2:**
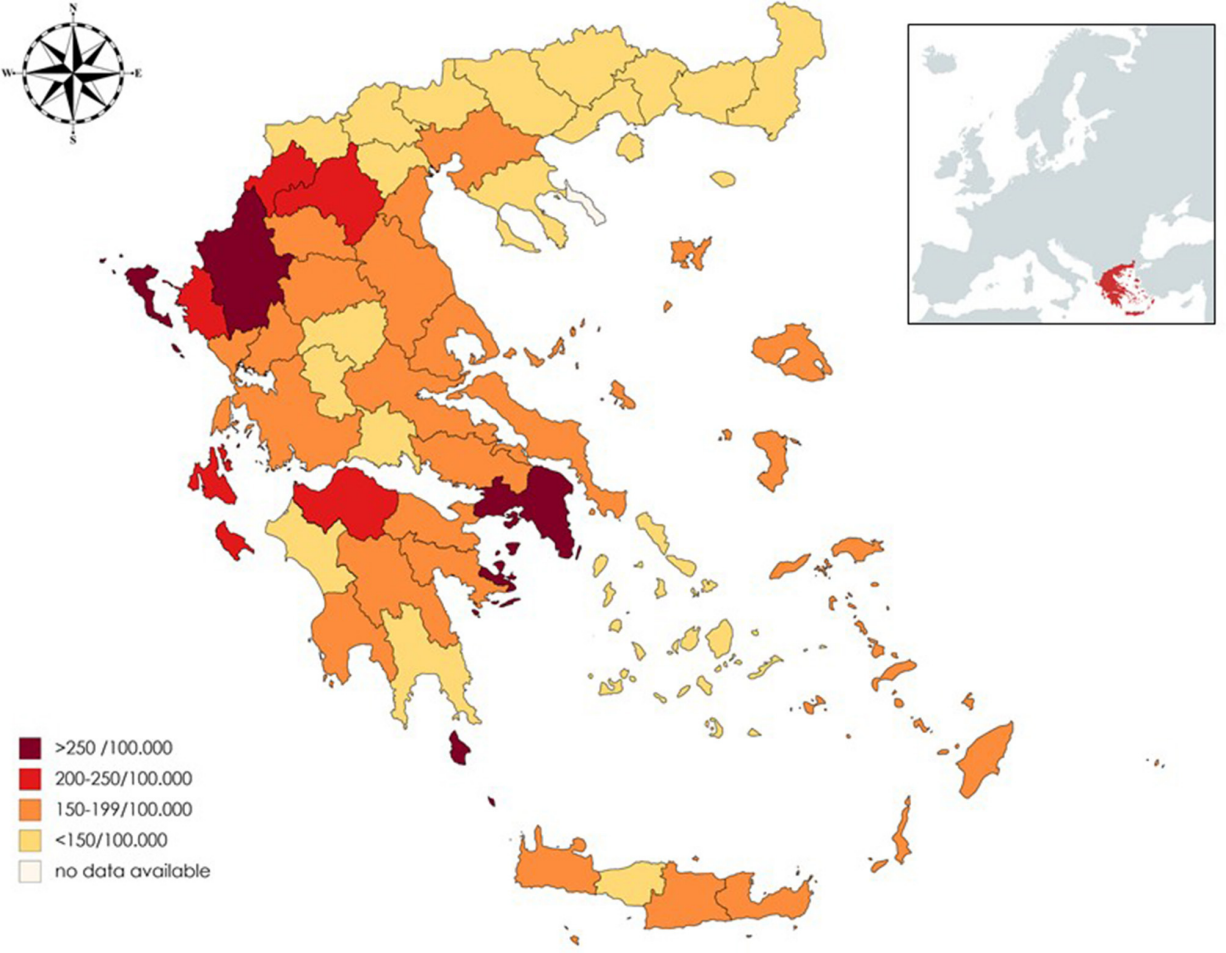
Map of Greece presenting prevalence of multiple sclerosis in each region.

**Figure 3:**
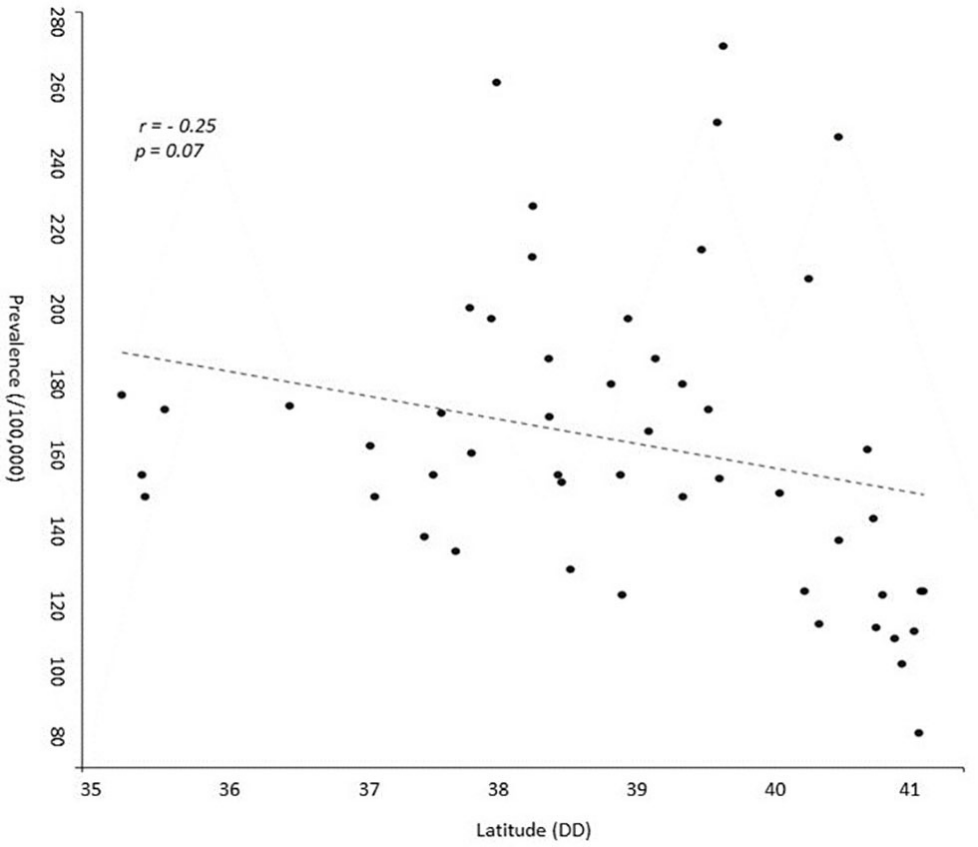
Scatterplot presenting prevalence of multiple sclerosis and latitude of each region of Greece. DD, Decimal Degrees.

According to our study results, 21,218 (13,994 or 65.8% females, sex ratio 1.93:1) individual cases of MS were identified. Mean age of the PwMS was 46.6 ± 13.5 (range 8–95; Figure 1). The 2-year period prevalence was 138.7 per 100,000 males (95% CI 138.4–139.0) and 253.6 per 100,000 for females (95% CI 253.3–254.1). Overall, 2-year period prevalence was calculated to 197.8 per 100,000 (95% CI 197.6–198.0). Higher prevalence was calculated in the 45–49 age group (371.3 per 100,000, 95% CI 370.2–372.4) for both males (250.5 per 100,000, 95% CI 249.1–251.9) and females (486.7 per 100,000, 95% CI 485.3–488.3; Table 2). Cases with age under 18 (*n* = 133, 81 or 60.9% females) accounted for 0.6% of the total identified MS cases. The estimated point prevalence on January 1, 2019, was 188.9 per 100,000 (95% CI 188.7–189.1). The higher prevalence rates were observed in Attica region (263.6 per 100,000, 95% CI 263.2–264.0) followed by regions of Western Greece, while the region of Rodopi in northern Greece presented the lowest prevalence rates (84.0 per 100,000, 95% CI 82.8–85.2; Table 3 and Figure 2). No north–south latitude gradient was observed (*r* = −0.25, *p* = 0.07; Figure 3).

**Table 2:** Two-year period administrative prevalence of multiple sclerosis according to age and sex.

**Age group**	**Male PwMS**	**Male population**	**Prevalence (/100,000)**	**95% CI**	**Female PwMS**	**Female population**	**Prevalence (/100.000)**	**95% CI**	**Total PwMS**	**Total population**	**Prevalence (/100,000)**	**95% CI**
0–4	0	2,39,732	0	–	0	2,26,944	0	–	0	4,66,676	0	–
5–9	2	2,71,053	0.7	[0.7–0.7]	1	2,56,980	0.3	[0.3–0.4]	3	5,28,033	0.5	[0.5–0.5]
10–14	16	2,78,937	5.7	[5.6–5.8]	21	2,63,429	7.9	[7.7–8.3]	37	5,42,366	6.8	[6.6–7.0]
15–19	91	2,83,450	32.1	[31.5–32.8]	148	2,62,687	56.3	[55.4–55.7]	239	5,46,137	43.7	[43.3–44.3]
20–24	265	2,86,472	92.5	[91.4–93.6]	437	2,65,850	164.3	[163.0–165.8]	702	5,52,322	127.0	[126.2–128.0]
25–29	415	2,89,329	143.4	[142.1–144.7]	850	2,79,643	303.9	[302.3–305.7]	1,265	5,68,972	222.3	[221.2–223.4]
30–34	638	3,04,326	209.6	[208.2–211.0]	1228	3,05,389	402.1	[400.4–403.8]	1,866	6,09,715	306.0	[304.8–307.2]
35–39	870	3,83,663	226.7	[225.5–228.1]	1754	3,82,507	458.5	[457.0–460.2]	2,624	7,66,170	342.4	[341.4–343.6]
40–44	952	3,97,530	239.4	[238.2–240.8]	1894	4,04,656	468.0	[466.6–469.6]	2,846	8,02,186	354.7	[353.8–355.8]
45–49	987	3,93,951	250.5	[249.1–251.9]	2006	4,12,102	486.7	[485.3–488.3]	2,993	8,06,053	371.3	[370.2–372.4]
50–54	916	3,76,235	243.4	[242.1–244.9]	1755	4,05,972	432.2	[430.8–433.8]	2,671	7,82,207	341.4	[340.4–341.5]
55–59	774	3,38,020	228.9	[227.6–230.4]	1494	3,80,563	392.5	[391.0–394.2]	2,268	7,18,583	315.6	[314.5–316.7]
60–64	549	3,16,380	173.5	[172.2–174.8]	1067	3,55,526	300.1	[298.6–301.6]	1,616	6,71,906	240.5	[239.5–241.5]
65–69	352	2,85,694	123.2	[122.0–124.4]	648	3,21,158	201.7	[200.4–203.2]	1,000	6,06,852	164.7	[163.9–165.7]
70–74	207	2,55,713	80.9	[79.9–82.1]	372	3,01,680	123.3	[122.1–124.5]	579	5,57,393	103.8	[103.1–104.7]
90–94	4	36,371	10.9	[10.6–11.3]	8	56,301	14.2	[13.3–15.2]	12	92,672	12.9	[12.2–13.6]
95–99	0	10,028	0	–	1	12,997	7.6	[6.3–9.4]	1	23,025	4.3	[3.5–5.2]
100+	0	3,814	0	–	0	5,518	0	–	0	9,332	0	-
Total	7,224	52,08,293	138.7	[138.4–139.0]	13994	55,16,306	253.6	[253.3–254.1]	21,218	107,245,99	197.8	[197.6–198.0]

*PwMS, people with multiple sclerosis; age is indicated in years*.

**Table 3:** Two-year administrative prevalence of multiple sclerosis in each region of Greece.

**Region**	**Number of PwMS**	**Total population**	**Prevalence (/100.000)**	**95% CI**
Drama	120	96,845	123.9	[121.8–126.0]
Evros	180	1,47,190	122.3	[120.6–124.0]
Kavala	147	1,33,391	110.2	[108.5–111.9]
Xanthi	138	1,11,631	123.6	[121.7–125.5]
Rodopi	93	1,10,666	84.0	[82.8–85.2]
Imathia	195	1,41,585	137.7	[135.9–139.5]
Thessaloniki	1,794	11,04,690	162.4	[161.7–163.1]
Kilkis	83	80,475	103.1	[101.0–105.2]
Pella	156	1,37,181	113.7	[112.0–115.4]
Pieria	163	1,31,879	123.6	[121.8–125.4]
Serres	189	1,67,374	112.9	[111.4–114.4]
Chalkidiki	127	1,10,593	114.8	[112.9–116.7]
Grevena	46	30,588	150.4	[146.4–154.4]
Kastoria	116	46,668	248.6	[244.7–252.5]
Kozani	294	1,40,233	209.7	[207.6–211.8]
Florina	71	49,519	143.4	[140.3–146.5]
Karditsa	158	1,05,403	149.9	[147.8–152.1]
Larissa	434	2,81,033	154.4	[153.1–155.7]
Kagnisia	366	2,03,162	180.2	[178.5–181.9]
Krikala	224	1,29,042	173.6	[171.5–175.7]
Krta	118	62,973	187.4	[184.4–190.5]
Thesprotia	98	45,064	217.5	[213.7–221.3]
Ioannina	459	1,67,696	273.7	[271.6–275.8]
Preveza	115	57,963	198.4	[195.2–201.7]
Kakinthos	80	39,737	201.3	[197.4–205.3]
Corfu	256	1,01,569	252.0	[249.3–254.7]
Cephalonia-Ithaka	89	38,718	229.9	[225.7–234.1]
Lefkada	43	23,845	180.3	[175.5–185.2]
Etoloakarnania	345	2,00,617	172.0	[170.4–173.7]
Khaia	645	2,98,996	215.7	[214.2–217.2]
Ilia	209	1,55,576	134.3	[132.6–136.0]
Viotia	190	1,22,256	155.4	[153.4–157.4]
Kvia	328	2,13,067	153.9	[152.4–155.4]
Kvritania	23	18,814	122.2	[117.6–127.0]
Fthiotida	248	1,59,387	155.6	[153.8–157.4]
Fokida	55	42,436	129.6	[126.4–132.8]
Argolida	167	96,564	172.9	[170.5–175.3]
Arkadia	127	81,680	155.5	[153.0–158.0]
Korinthia	294	1,47,739	199.0	[197.0–201.0]
Lakonia	135	90,368	149.4	[147.1–151.7]
Messinia	259	1,58,096	163.8	[162.0–165.6]
Kttica	9,865	37,42,235	263.6	[263.2–264.0]
Lesvos-Limnos	192	1,14,805	167.2	[165.1–169.4]
Samos-ikaria	78	48,238	161.7	[158.4–165.0]
Chios	109	58,055	187.8	[184.6–191.0]
Dodecanese	378	2,17,241	174.0	[172.4–175.6]
Cyclades	175	1,26,786	138.0	[136.1–139.9]
Heraklion	489	3,13,766	155.8	[154.5–157.1]
Lasithi	131	74,006	177.0	[174.3–179.8]
Rethymno	131	87,582	149.6	[147.3–152.0]
Chania	277	1,59,576	173.6	[171.7–175.5]
Unknown	16			

*PwMS, people with multiple sclerosis*.

**Figure 4:**
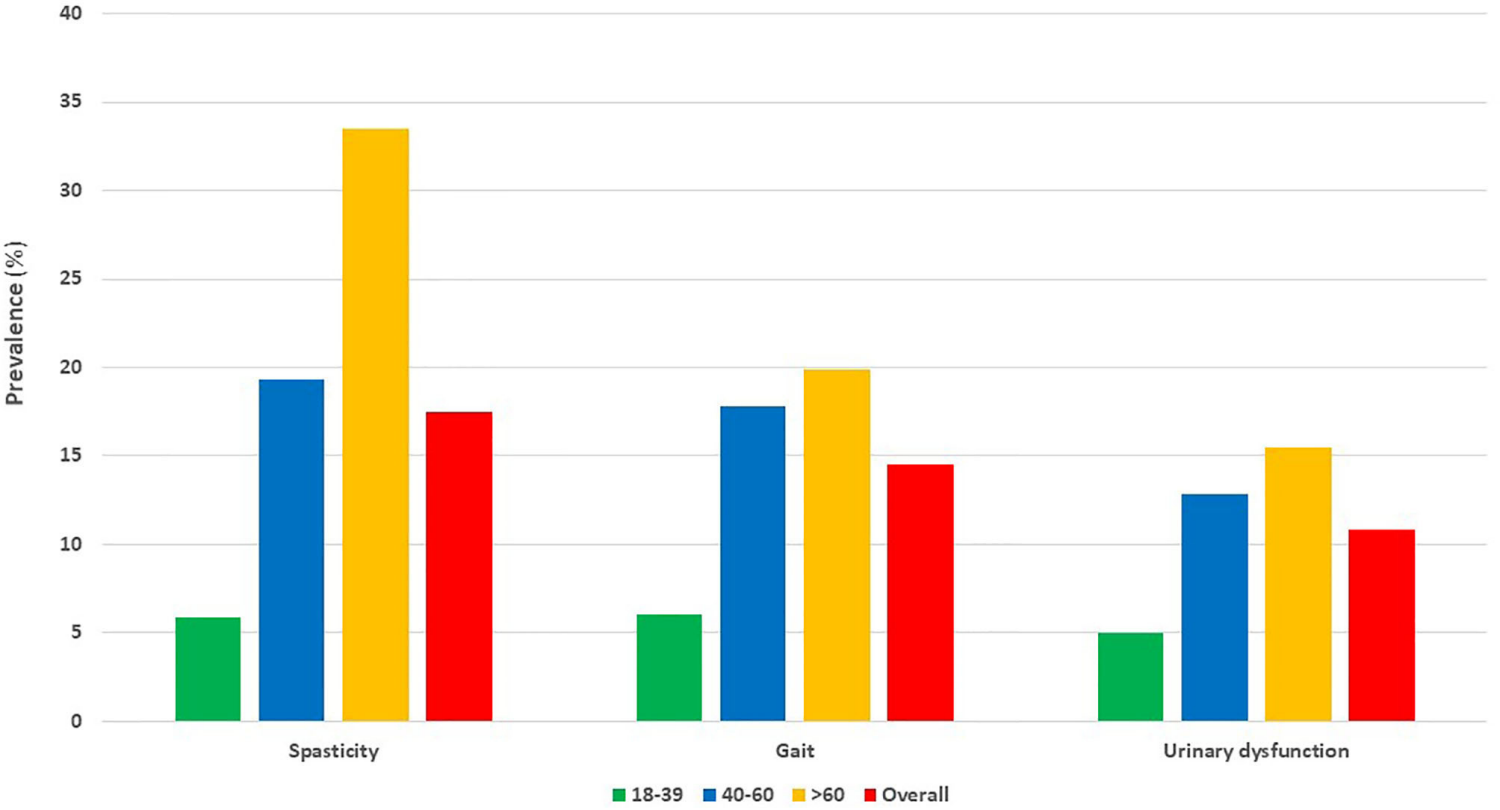
Most commonly prescribed drugs for symptomatic conditions in adult people with multiple sclerosis, according to age. Bars represent percentage (%) of adult people with multiple sclerosis for different age groups. PwMS, people with multiple sclerosis; age is indicated in years.

Regarding treatment, 15,520 (73.1 %) PwMS were treated at least once with a prescribed DMD during the study period. The most frequently prescribed drugs for symptomatic treatment were anti-spasticity agents (17.5%), followed by fampridine (14.5%) and urinary antispasmodics (10.8%) (Figure 4).

## Discussion

Prevalence of MS is increasing worldwide (16). This phenomenon may be attributed to improved access to health professionals and magnetic resonance imaging (17). In addition, current diagnostic criteria enable an earlier diagnosis of the disease (18, 19). Early treatment initiation is suggested to improve long-term disability outcomes and potentially affect mortality due to MS related complications (20, 21).

According to our study results and previous epidemiological ones, prevalence of MS in Greece has substantially increased within a decade. This might be due to increased access to healthcare professionals; Greece presents one of the highest rates of physicians per population (6.3 per 1,000 people in 2013) (22). Moreover, nowadays, uninsured PwMS have unrestricted access to a public healthcare system and DMDs, and therefore they can be recorded in the digital prescription database of IDIKA S.A. In addition, MS patient organizations may also have contributed to the raising of public awareness regarding the disease, by actively organizing events during the last years.

Although sex ratio in this study was in accordance with international studies regarding MS (16), a latitudinal gradient was not detected, though, heterogeneity between regions was observed. Attica prefecture exhibits the higher prevalence of PwMS (263 per 100,000). Athens, which is the capital and largest city in Greece, is located in Attica. Previous studies have suggested that MS prevalence is higher in urban areas, especially in large cities (15, 23, 24). Whether risk factors associated with urbanization, such as air pollution and/or more direct access to specialized healthcare, contribute to these results remains to be elucidated (25). High prevalence rates were also calculated for Western Greece, while lower prevalence rates were exhibited in Northern Greece. The existence of environmental or other risk factors in these areas should further be explored. Study results are in accordance with current literature suggesting no obvious north–south gradient of MS prevalence within a country (26, 27). This might be due to the limited latitude extent of Greece and other studied countries. In addition, in western countries, latitudinal difference may not be observed perhaps due to the reported homogenization of lifestyle habits (28).

For both sexes, higher prevalence was calculated in the 45–49 age group. These results are in accordance to other studies that report similar age distribution among PwMS (24, 29–33). Moreover, change in age distribution over time toward gradually increasing age at prevalence peak has been proposed as an indicator of reduced mortality for PwMS (29). The present study provides a cross-sectional estimation of MS prevalence in Greece based on a 2-year period and does not include consecutive estimations of MS prevalence over several decades. Therefore, this study could not explore changes in age distribution over time. However, by taking into account overall improvements in parameters of quality of life and access to health services and DMDs in Greece over the last decades, it is likely to consider the observed 45–49 age peak as an indicator of improved survival for PwMS in Greece. Furthermore, the nationwide availability of MRI during the last 25 years may have contributed to this finding.

The use of administrative data and registries rather than extrapolations from local medical records has led to a recent reconsideration of MS prevalence in many countries. The number of PwMS seems to be much higher than expected worldwide. In Italy, a recent study reported prevalence of MS 208.7 per 100,000 in 2017 in the region of Tuscany (34), using administrative data. A meta-analysis based on previously published relevant studies in Italy estimated current prevalence to 176 per 100,000 (35). In Norway, a nationwide study estimated a prevalence of 203 per 100,000 in 2012 (27). In the same year, using nationwide administrative data, prevalence of MS in France was upwards revised to 151.2 per 100,000 inhabitants, although researchers stated that at least 15% of the actual PwMS may have not been recorded (36). In Bavaria, Germany, prevalence was increased from 171 per 100,000 in 2006 to 277 per 100,000 in 2015, according to a study using health insurance data (24). In the United States, the use of health claims data has led to a reconsideration of MS prevalence which was estimated to be 309.2 per 100,000 in 2010 (37).

Onset of MS before the age of 18 varies between 2 and 4% depending on study (38). The age cutoff used for pediatric MS is the age of 16; however, the age 18 is also used by some researchers (39). The diagnosis of MS in youngsters is challenging since various conditions may present with similar clinical and radiological findings (40). The juvenile/adolescent cases identified in the current study are less than expected. This finding may reflect the special precautions taken by physicians in order to set a definite MS diagnosis at young age. Moreover, 94.7% of PwMS under 18 years of age had received a DMD, but only rarely were they treated with drugs for symptomatic conditions, a finding that further underlines an overall reluctance of physicians to prescribe several types of medication in patients of this age group. In addition, further investigation is needed in order to explore whether access to specialized physicians is available for this specific population.

This study used prescription data of almost the entire Greek population, rather than extrapolating results from regional registries; thus, a more accurate MS prevalence in this country may have been estimated, at least with respect to adult PwMS. However, there is some weakness in our study for a number of reasons: Data about the level of disability, disease duration, and type could not be extracted. In addition, since no ICD-10 classification for clinically isolated syndrome (CIS) exists, people with CIS receiving DMDs may have been misclassified as PwMS. However, as the revisions of the McDonald criteria have enabled an earlier MS diagnosis and patients with a CIS diagnosis have substantially decreased (17, 19), this discrepancy may have not significantly modified the estimated MS prevalence.

According to our study, 73.1% of the studied population has received a DMD. This percentage may also be underestimated, although not to a major degree, since patients participating in pre-approval clinical trials have not been recorded as receiving a DMD. Moreover, a proportion of PwMS treated with intravenous monoclonal antibodies may have not been recorded as PwMS under DMD treatment, due to the exclusion of some national hospitals' in-house treatments from this prescription database. However, most of these cases were included on the basis of their prescriptions for symptomatic treatment. Nevertheless, the percentage of PwMS under DMD was estimated as particularly high and this could be attributed to the fact that Greek adult PwMS have unlimited access to DMDs, regardless of age and type of insurance. Furthermore, PwMS not receiving any prescription for DMD or symptomatic treatment during this 2-year period were not captured by this study, and this may have affected the estimation of the percentage of PwMS under DMDs. Data regarding PwMS who may have passed away during the study period were not taken into account. PwMS over 60 years old were more frequently treated with prescribed drugs for symptomatic treatment. This finding could be attributed to the impact of long disease duration and aging in the accumulation of disability in MS and the higher prevalence of the progressive forms of the disease in this age group.

Various algorithms have been validated in order to identify MS cases using secondary administrative data (36, 37, 41–43). Due to the variations of health policies, prescription systems, and registries amongst countries, these algorithms are not universally applicable on all administrative datasets, apart from those that are validated. In Greece, the lack of a nationwide registry of hospital admissions and digital medical records renders the validation of such an algorithm challenging. Therefore, our study does not have the strength to explore data validity in a quality degree of a national registry.

Incidence estimates may better reflect differences in chronic disease rates (2); however, when a short time period is studied, incidence may be overestimated. Cases may be misclassified as new if no data regarding the previous years exist^4^. Given that the period studied was short, we did not proceed to the calculation of current incidence of MS.

In lack of a nationwide registry for MS, we hereby provide for the first time an estimation of the current prevalence of MS in Greece, on the basis of the national administrative database for drug prescription. Regional variations of the MS prevalence in Greece were elucidated, and these results underscore the need for a thorough evaluation of the underlying factors (genetic, environmental, etc.) that may account for the observed differences. In spite of the methodological limitations stemming from the use of administrative data, our results verify increased MS prevalence, relatively to previous reports, in accordance to observations also made for other European populations. Moreover, the results of the present study point toward specific needs for optimization of the allocation of resources regarding MS in Greece and highlight the necessity of a national registry for PwMS that will include detailed and validated physician-reported clinical parameters. A nationwide registry for PwMS is currently under construction and is about to run a pilot phase soon. This registry is expected to enable further investigation of the regional MS prevalence heterogeneity with regards to genetic variances and access to healthcare.

## Data Availability Statement

The datasets presented in this article are not readily available because raw data used in this study are property of the Greek Ministry of Health. Requests to access the datasets should be directed to https://www.moh.gov.gr/.

## Ethics Statement

The studies involving human participants were reviewed and approved by Ministry of Health, Greece. Written informed consent from the participants' legal guardian/next of kin was not required to participate in this study in accordance with the national legislation and the institutional requirements.

## Author Contributions

CB, EG, GP, DK, and NG: study concept, design, and supervision. CB, EG, MB, and IN: acquisition of data. CB, SS, TM, and DTsak: extraction of data and statistical analysis. EK, CB, EG, NG, ET, CP, and DTsal: analysis and interpretation. CB, EG, and MB: drafting of the manuscript. All co-authors: critical revision of the manuscript for important intellectual content and final approval of the version.

## Conflict of Interest

The authors declare that the research was conducted in the absence of any commercial or financial relationships that could be construed as a potential conflict of interest.

## Footnotes

^1^http://www.idika.gr

^2^*Hellenic Statistical Authority*. Available online at https://www.statistics.gr

^3^www.eopyy.gov.gr

^4^https://www.nato.int/nato_static_fl2014/assets/pdf/pdf_2019_06/20190625_PR2019-069-EN.pdf

^5^https://www.e-prescription.gr
